# Fabrication and Characterization of a Tunable In-plane Resonator with Low Driving Voltage

**DOI:** 10.3390/s90302062

**Published:** 2009-03-18

**Authors:** Pin-Hsu Kao, Ching-Liang Dai, Cheng-Chih Hsu, Chi-Yuan Lee

**Affiliations:** 1 Department of Mechanical Engineering, National Chung Hsing University, Taichung, 402 Taiwan, ROC; E-Mail: d9461402@mail.nchu.edu.tw; 2 Department of Electro-Optical Engineering, Yuan Ze University, Taoyuan, 320 Taiwan, ROC; E-Mail: cchsu@saturn.yzu.edu.tw; 3 Department of Mechanical Engineering, Yuan Ze Fuel Cell Center, Yuan Ze University, Taoyuan, 320 Taiwan, ROC; E-Mail: cylee@saturn.yzu.edu.tw

**Keywords:** Micromechanical tunable resonators, Low driving voltage, CMOS-MEMS

## Abstract

This study presents the fabrication and characterization of a micromechanical tunable in-plane resonator. The resonator is manufactured using the commercial 0.35 μm complementary metal oxide semiconductor (CMOS) process. The resonator is made of aluminum, and the sacrificial layer is silicon dioxide. The post-process involves only one maskless etching step using an etchant to remove the sacrificial layer. The resonator includes three parts: a driving part to provide a driving force, a sensing part that is used to detect a change in capacitance when the resonator is vibrating, and a tuning part that changes the resonant frequency of the resonator. The main advantages of the tunable resonator are a low driving voltage and compatibility with the CMOS process. The resonant frequency of the resonator can be changed upon applying a dc voltage to the tuning part. To reduce the driving voltage, the driving part is designed as comb-finger rows. Experimental results show that the resonator has a resonant frequency of about 183 kHz and a driving voltage of 10 V; the resonant frequency increases 14 kHz when a tuning voltage of 30 V is applied. The resonator has a maximum frequency–tuning ratio of 7.6%.

## 1. Introduction

With the advances in science and technology, industrial communication has become inseparable from daily life. According to a report from the National Communications Commission in Taiwan, the penetration rate of the mobile phone in Taiwan was only 6.9% in 1997, and grew to 105.8% in 2007. As the size of these devices continues to decrease, a filter is needed to control the quality of communication. Filters have become an important electronic component within the electronic industry in order to keep pace with the developments that are being made rapidly. Micromechanical resonators can be used as filters for wireless communication applications [1,2]. The main advantage of micromechanical resonators is high quality factor (Q-factor).

Many studies have recently used microelectromechanical systems (MEMS) technology to fabricate micromechanical resonators. For example, Yao *et al*. [3] proposed a single-crystal silicon (SCS) micro tunable resonator fabricated by reactive ion etch (RIE) process and microfabrication technique, which the tunable resonator had a driving voltage of 35 V, a Q-factor of 10,000 in vacuum and a tuning range of 60 kHz. Patil *et al.* [4] fabricated a thin film silicon microbridge resonator on a glass substrate using surface micromachining. The structural material of the resonator was a bilayer of aluminum and phosphorus-doped hydrogenated amorphous silicon, and the sacrificial material was polynorbornene. The resonant frequency and Q-factor of the microbridge resonator were about 4.4 MHz and 450, respectively. Zhang *et al.* [5] utilized surface micromachining to manufacture a bridge microresonator with a conductive polymer blend as the structural layer, and the sacrificial layer was aluminum. The resonant frequency of the resonator was about 3 MHz, and the Q-factor was 100 in vacuum. The polycrystalline 3C silicon carbide (polySiC) lateral resonators, proposed by Roy *et al.* [6], were made by a three-mask surface micromachining process using silicon dioxide, polysilicon, and nickel as the isolation, sacrificial, and contact metallization layers, respectively. The driving voltage and Q-factor of the ploySiC resonators at atmospheric pressure were 40–170 V and 148, respectively. The polySiC resonators had a driving voltage of about 1 V and a Q-factor of over 100000 under high vacuum condition (< 10-5 torr). Dai *et al.* [7] presented a tunable resonator fabricated by using the CMOS-MEMS process. This resonator could be tuned when applying a dc bias to a comb of linearly varied finger length; the driving voltage was approximately 20 V. Using the same process, Dai *et al.* [8] manufactured a plane resonator, and the driving voltage and the resonant frequency were 60 V and 39.5 MHz, respectively.

The CMOS-MEMS technique [9–11] is the use of a commercial CMOS process to fabricate MEMS devices. Micro devices manufactured by the CMOS-MEMS technique usually need some post-processing to release the suspended structures or coat the functional films. For instance, the suspended structures of the CMOS-MEMS pressure sensors [12] were released by a wet etching post-process, and the film sensitive to ammonia [13] was coated by a post-process. In this work, we utilized the CMOS-MEMS technique to make the tunable in-plane resonator. The commercial 0.35 μm CMOS process of the Taiwan Semiconductor Manufacturing Company (TSMC) was used to fabricate the micromechanical resonator. The post-process employed a wet etching treatment to remove the sacrificial layer and release the suspended structures in the resonator. The tunable resonator contains three parts: the driving, sensing, and tuning parts. The sensing part senses a change in capacitance when a voltage is applied to the driving part, and the resonant frequency of the resonator can be tuned by the tuning part. Experimental results depict that the resonant frequency was about 183 kHz, and increased by 14 kHz when a tuning voltage of 30 V was applied.

## 2. Design and Simulation

**Figure 1.**
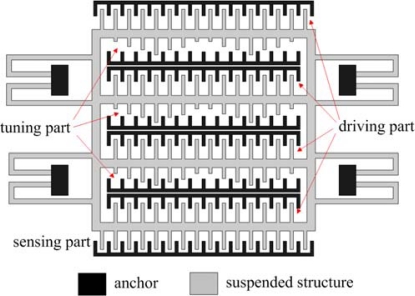
Schematic structure of the tunable resonator.

Figure 1 illustrates the structures of the micromechanical resonator, which includes a driving part, a sensing part and a turning part. The sensing and driving parts have a constant-length comb configuration that consists of the moveable and fixed combs. The driving voltage depends on the number of comb-finger of driving part and the stiffness of supported beams. In order to reduce the driving voltage, the driving part of the resonator is designed with four comb-finger rows. There are eight support beams arranged symmetrically. Each beam is 260 μm long, 2 μm wide and 2.6 μm thick, and it is fixed to the 20×40 μm^2^ anchor. The resonator is a suspended membrane with a thickness of 5.8 μm; the gap between the membrane and the substrate is approximately 1.3 μm. The area of the resonator is about 460×260 μm^2^.

The resonator is actuated by the electrostatic force. When applying an ac voltage, *V_s_*(*t*)=*V_0_* sin*ωt*, to the driving part, the driving force produced by the comb-fingers of the driving part can be expressed as [14],

(1) $$F_{d}\;(t)=F_{0}\text{sin}\omega t$$

and

(2) $$F_{0}=\frac{n\varepsilon t_{h}V_{0}^{2}}{2d}$$

where *n* represents the number of fingers in the driving-comb; *ε* is the permittivity constant of air, *t_h_* is the comb thickness and *d* is the inter-finger gap of the comb. The equation of motion of the micromechanical tunable resonator is given by,

(3) $$m\ddot{x}+c\dot{x}+\mathrm{kx}=F_{d}\;(t)$$

where *m* represents the mass of the resonator; *c* is the damp; *k* is the stiffness of the resonator and *x* is the dynamic displacement of the resonator. The particular solution of Equation (3) can be expressed as [15],

(4) x(t)=Xsin(ωt−ϕ)

and

(5) $$X=\frac{\frac{F_{0}}{k}}{\sqrt{{\left(1-r^{2}\right)}^{2}+{\left(2\varsigma r\right)}^{2}}}$$

where *X* and *ϕ* are the amplitude and phase angle of the response, respectively; *r* is the frequency ratio and *r*=*ω*/*ω_n_*; *ζ* is the damping ratio and *ζ*=*c*/*2mω_n_*; *ω_n_* is the natural frequency of the resonator. The maximum amplitude occurs when $r=\sqrt{1-2\varsigma^{2}}$ [15]. Substituting Equation (2) and $r=\sqrt{1-2\varsigma^{2}}$ into Equation (5), the maximum amplitude of the micromechanical tunable resonator can be obtained,

(6) $$X_{\text{max}}=\frac{n\varepsilon t_{h}V_{0}^{2}}{4\mathrm{dk}\varsigma \sqrt{1-\varsigma^{2}}}$$

**Figure 2.**
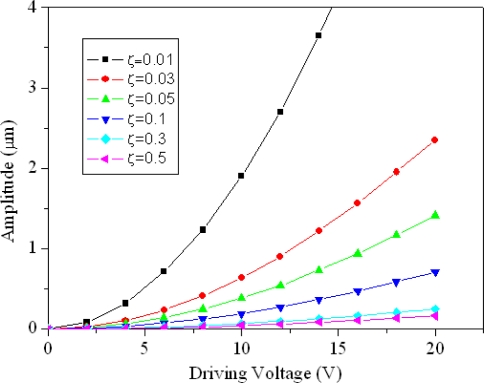
Maximum amplitude of the tunable resonator at different damping ratios.

In the design, the stiffness of the resonator, *k*, is about 17 N/m, and the number of fingers in the driving-comb, *n*, is 264. The inter-finger gap of the comb, *d*, is 1 μm and the comb thickness, *t_h_*, is 5.8 μm. Figure 2 shows the maximum amplitude of the micromechanical tunable resonator with different damping ratios, which is evaluated by Equation (6). In addition to the geometric shape of the resonator, the maximum amplitude of the resonator depends on the driving voltage and the damping ratio.

**Figure 3.**
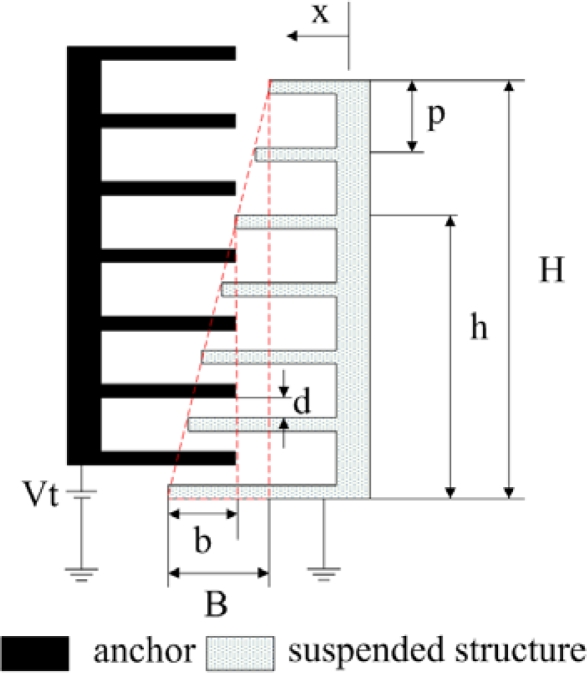
Tuning-comb of the tunable resonator.

Figure 3 illustrates the geometry of the tuning part in the resonator, which contains the moveable and fixed combs. The moveable comb of the turning part is designed as linearly varied finger length. The resonant frequency of the micromechanical tunable resonator is given by [7],

(7) $$f=\frac{1}{2\pi }\sqrt{\frac{k_{\mathrm{eff}}}{m}}$$

and

(8) $$k_{\mathrm{eff}}=k+\frac{N\varepsilon {\mathrm{Ht}}_{h}\;\left(b+x\right)}{2\mathrm{Bpdx}}V_{t}^{2}$$

where *k_eff_* represents the effective stiffness of the resonator; *N* is the number of fingers in the tuning-comb; *ε* is the permittivity constant of air; *H* and *B* are the width and height of the tuning-comb triangle, respectively; *t_h_* is the comb thickness; *V_t_* is the tuning voltage of the tuning part; *p* is the pitch of the tuning-comb fingers; *d* is inter-finger gap of the comb; *x* is the displacement of the moveable structure; and *b* is the overlapping length of the comb finger, as shown Figure 3. In accordance with Equation (7), we know that the resonant frequency of the resonator changes as the effective stiffness of the resonator varies. According to Equation (8), the effective stiffness of the resonator depends on the geometric shape, tuning-comb number, and tuning voltage of the tuning part. Therefore, the resonant frequency of the resonator can be controlled by the tuning part. The effective stiffness increases when applying a tuning voltage to the resonator, so that the resonant frequency of the resonator is increased.

**Figure 4.**
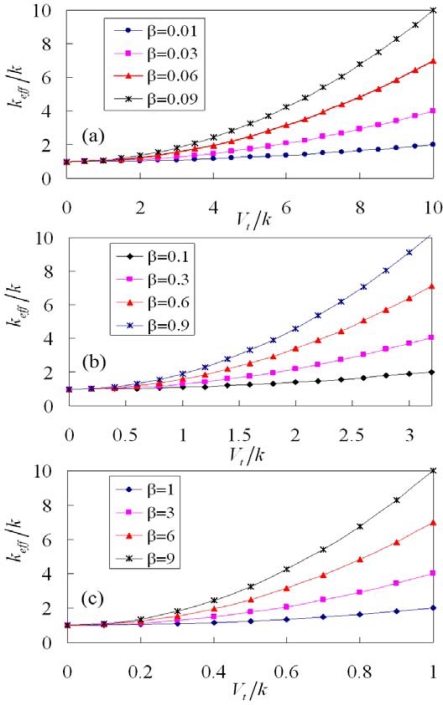
Stiffness ratio of the tunable resonator with different shape factors.

In order to characterize the relation between the effective stiffness, geometric shape, tuning voltage of the tunable resonator, Equation 8 is arranged as,

(9) $$\frac{k_{\mathrm{eff}}}{k}=1+\beta {\left(\frac{V_{t}}{k}\right)}^{2}$$

and

(10) $$\beta =\frac{N\varepsilon {\mathrm{Ht}}_{h}k\;\left(b+x\right)}{2\mathrm{Bpdx}}$$

where *k_eff_*/*k* denotes the stiffness ratio that is the ratio of the effective stiffness and the stiffness in the resonator; *β* is the shape factor related to the geometric shape of the tuning part and the beams and the unit is N^2^/V^2^·m^2^; *V_t_*/*k* is the tuning voltage to stiffness (TVS) ratio that represents the ratio of the tuning voltage and the stiffness in the resonator and the unit is V·m/N. According to Equation (9), the stiffness ratio of the resonator with different shape factors is plotted in Figure 4.

The stiffness ratio, *k_eff_*/*k*, is unity when the tuning voltage is zero; this means that the effective stiffness of the resonator is equal to its stiffness when without tuning driving. As shown in Figure 4(a), the value of shape factor, *β*, is changed from 0.01 to 0.09. The stiffness ratio increases as the shape factor increases under the same tuning voltage. In addition to the shape factor, the stiffness ratio increases as the TVS ratio increases. As shown in Figure 4(b), the value of shape factor is varied from 0.1 to 0.9. The results show that the stiffness ratio increases as the shape factor and the TVS increase. Under the same stiffness ratio, the TVS ratio (*V_t_*/*k*) in Figure 4(b) is smaller than that in Figure 4(a) because the shape factors in Figure 4(b) have a larger value. For instance, suppose that the resonator requires achieving the stiffness ratio of 10, the TVS ratios are 10 (Figure 4(a)) and 3.1 (Figure 4(b)) when the shape factors adopt 0.09 and 0.9, respectively. This means that the TVS ratio can be reduced by using the increment of shape factor. As shown in Figure 4(c), the value of shape factor is changed from 1 to 9. The trends of curves in Figure 4(c) are similar to that in Figure 4(a) and 4(b). The results show that the TVS ratio corresponding to the stiffness ratio of 10 at the shape factor of 9 is unity.

**Figure 5.**
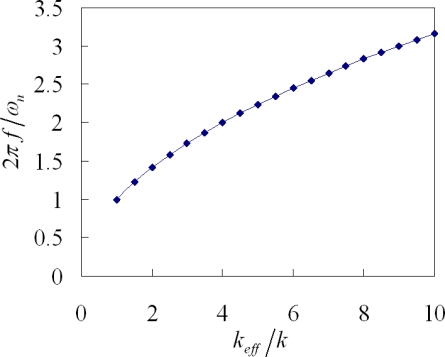
Frequency ratio vs. stiffness ratio.

The resonant frequency of the tunable resonator depends on its stiffness ratio. Equation (7) can be written as,

(11) $$\frac{2\pi f}{\omega_{n}}=\sqrt{\frac{k_{\mathrm{eff}}}{k}}$$

where 2*πf*/*ω_n_* is the frequency ratio that is the ratio of the resonant frequency and the natural frequency ( $\omega_{n}=\sqrt{k/m}$) in the resonator; and *k_eff_*/*k* is the stiffness ratio of the resonator. According to Equation (11), we know that the frequency ratio is proportional to the square of the stiffness ratio. Figure 5 shows the frequency ratio of the tunable resonator, which is computed by Equation (11). The results depict that the frequency ratios of the resonator are 1 and 3.16 corresponding to the stiffness ratios of 1 and 10, respectively.

The frequency ratio of the resonator relies on its tuning voltage to stiffness ratio. Substituting Equation (9) into Equation (11), the frequency ratio of the tunable resonator can be expressed as,

(12) $$\frac{2\pi f}{\omega_{n}}=\sqrt{1+\beta {\left(\frac{V_{t}}{k}\right)}^{2}}$$

**Figure 6.**
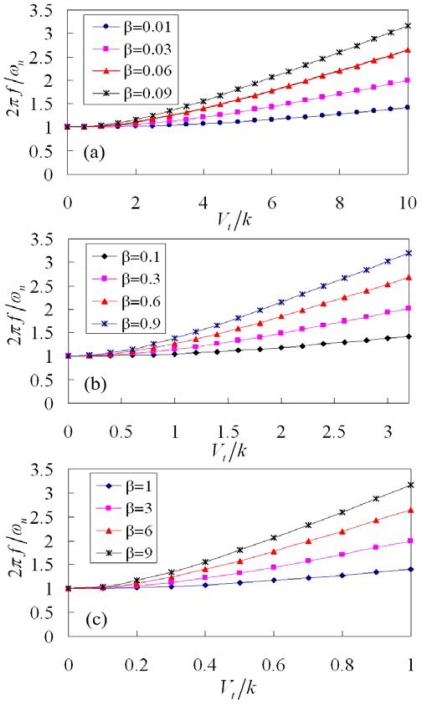
Frequency ratio of the tunable resonator with different shape factors.

Figure 6 illustrates the frequency ratio of the tunable resonator with different shape factors, which is evaluated by Equation (12). The values of shape factor in Figure 6(a), (b) and (c) are 0.01–0.09, 0.1–0.9 and 1–9, respectively. The frequency ratio of the resonator is unity when the tuning voltage, *V_t_*, is zero; this means that the resonant frequency of the resonator is equal to its natural frequency when without tuning driving. The trends of curves in Figure 6(b) and (c) are similar to that in Figure 6(a). The scales of y-axis (frequency ratio) in Figures 6(a)–(c) are the same, and the scales of x-axis (*V_t_*/*k*) are different. Under the same frequency ratio, the TVS ratio in Figure 6(b) is smaller than that in Figure 6(a) due to the shape factors in Figure 6(b) have a larger value. For example, suppose that the resonator requires achieving the frequency ratio of 1.4, the TVS ratios are 10 (Figure 6(a)), 3.1 (Figure 6(b)) and 1 (Figure 6(c)) when the shape factors adopt 0.01, 0.1 and 1, respectively. Therefore, the frequency ratio increases as the TVS ratio increases. In addition to the TVS ratio, the frequency ratio increases as the shape factor increases at the same tuning voltage.

The resonant frequency of the tunable resonator by Equation (12) can be written as,

(13) $$f=\frac{\omega_{n}}{2\pi }\sqrt{1+\beta {\left(\frac{V_{t}}{k}\right)}^{2}}$$

By Equations (12) and (13), we know that the resonant frequency of the resonator can be obtained if the frequency ratio, 2*πf*/*ω_n_*, is given. The resonant frequency of the resonator relies on the natural frequency, the shape factor and the TVS ratio. The shape factor (Equation 10) is proportional to the parameters of *N*, *H*, *ε*, *b*, *t_h_* and *k*, and is inverse proportional to the parameters of *B*, *p* and *d*. If the design of the tunable resonator needs a large shape factor, then the parameters of *N*, *H*, *ε*, *b*, *t_h_* and *k* should be increased and the parameters of *B*, *p* and *d* should be reduced. As shown in Figure 6, the shape factor should consider adopting a larger value if wishing to obtain a low TVS ratio. The procedure of design includes: (1) the specification of the resonator must be determined before the design of the resonator; (2) the frequency ratio of the resonator is obtained according to its specification; (3) the TVS ratio and the shape factor of the resonator can be found in accordance with its frequency ratio and the results in Figure 6; (4) the geometric shape and stiffness of the resonator are evaluated by means of the shape factor (Equation 10).

**Figure 7.**
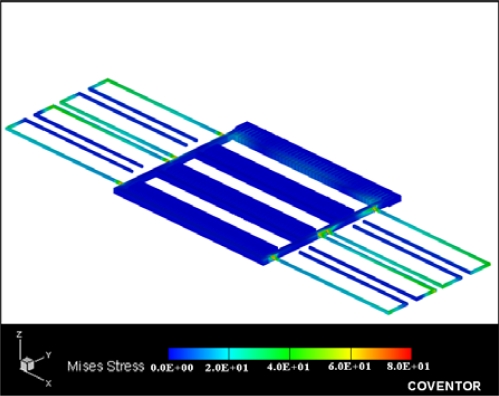
Stress distribution of the resonator.

The finite element method (FEM) software, Coventor Ware, is employed to simulate the stress distribution of the micromechanical resonator. Figure 7 presents the stress distribution of the simulated resonator under a driving voltage of 30 V. The maximum stress of the resonator is 80 MPa, which is located at the fixed end of the beams. The structure is an elastic deformation if the maximum stress is below the yield strength of its material. The resonator is made of aluminum and tungsten. The yield strength of aluminum and tungsten is about 135 MPa [16] and 450 MPa [17], respectively. Since the maximum stress of the resonator is less than the yield strength of aluminum, the resonator can be operated in an elastic range under a driving voltage of 30 V.

## 3. Fabrication of the Tunable Resonator

**Figure 8.**
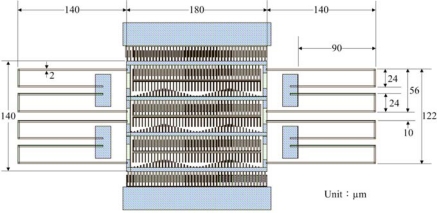
Layout of the tunable resonator.

The resonator is manufactured using the commercial 0.35 μm CMOS process. Figure 8 illustrates the layout of the micromechanical tunable resonator.

**Figure 9.**
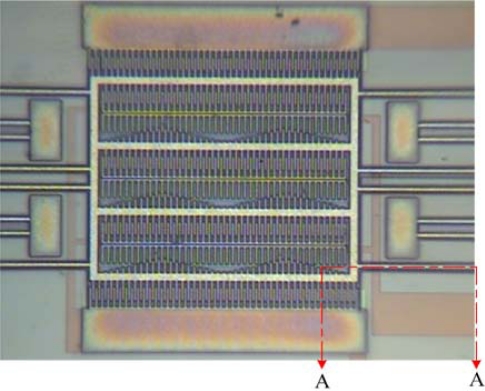
Optical image of the tunable resonator after completion of the CMOS process.

**Figure 10.**
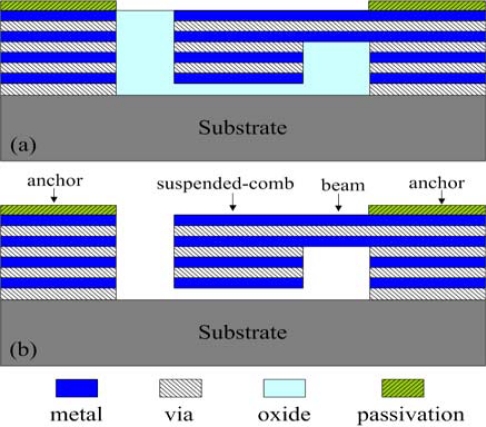
A-A cross section of the tunable resonator; (a) after completion of the CMOS process, (b) after completion of the post-process.

According to the layout of the resonator, TSMC employs the CMOS process to fabricate the resonator. Figure 9 shows the optical image of the tunable resonator after completion of the CMOS process. The A-A cross section of the resonator (Figure 9) after completion of the CMOS process is shown in Figure 10(a). The structural layers of the resonator are the metal and via layers, and the materials of metal and via layers are aluminum and tungsten, respectively.

**Figure 11.**
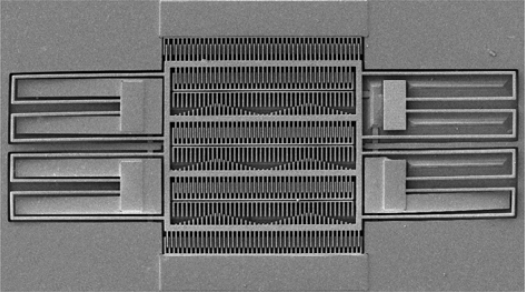
SEM image of the tunable resonator after completion of the post-process.

The sacrificial layer of the resonator is the silicon dioxide layer. The structural layers link up the sacrificial layer after the CMOS process. In order to obtain the suspended structural layers, the resonator requires a post-CMOS process to remove the sacrificial layer. The post-process utilizes an etchant (Silox) [18] to remove the sacrificial layer, and to obtain the suspended structures of the resonator. Figure 10(b) displays the cross section of the tunable resonator after completion the post-process. Figure 11 demonstrates the scanning electron microscope (SEM) image of the resonator after completion of the post-process.

## 4. Results and Discussion

**Figure 12.**
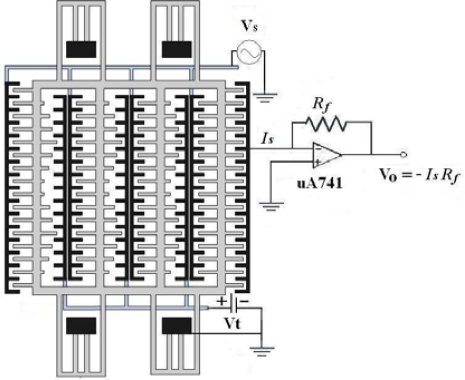
Sensing circuitry of the resonator.

The sensing part of the resonator generated a change in capacitance when applying a driving voltage to the driving part. Figure 12 illustrates the sensing circuitry of the tunable resonator [19].

**Figure 13.**
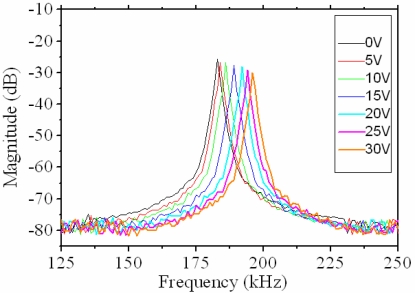
Frequency response of the tunable resonator at different tuning voltages.

The sensing circuitry was used to convert the capacitance variation of the sensing part in the resonator into the output voltage. The experimental setup for measuring the frequency response of the resonator included a power supply, a function generator, and a spectrum analyzer. The power supply provided a dc bias voltage to the tuning part, and the function generator supplied a swept sine voltage of 10 V to the driving part in the resonator to actuate the moveable comb. The spectrum analyzer was utilized to measure the frequency response of the resonator. Figure 13 displays the frequency response of the resonator with different tuning voltages. The measured result showed that the resonant frequency of the resonator without the tuning voltage was 183 kHz.

The Q-factor of the resonator is given by,

(14) $$Q=\frac{f_{o}}{{\left(\Delta f\right)}_{3\mathrm{dB}}}$$

where *f_0_* is the resonant frequency, and (Δ*f*)_3_*_dB_* is the -3dB peak width. The Q-factor of the resonator was approximately 400, which was calculated by Equation (14). When the different tuning voltages were applied to the tuning part, the resonant frequency of the resonator changed. As shown in Figure 13, the experimental results depicted that the resonant frequency of the resonator was 184, 185, 187, 190, 193 and 197 kHz upon applying the tuning voltage of 5, 10, 15, 20, 25 and 30 V, respectively.

**Figure 14.**
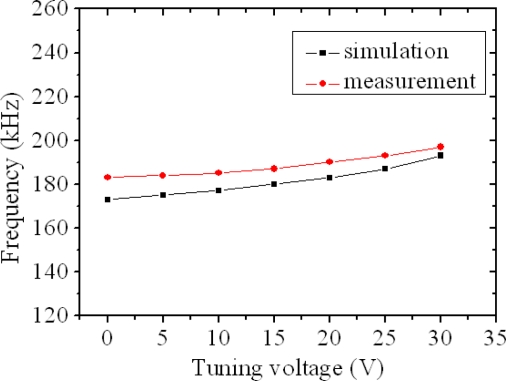
Resonant frequency vs. tuning voltage.

The resonator burned when the tuning voltage was over 30 V. The simulated resonance frequency of the resonator with different tuning voltages is shown in Figure 14, which is calculated by Equation (13). The simulation presented that the resonator without the tuning voltage had a resonant frequency of 173 kHz, and its resonant frequency changed to 173, 177, 180, 183, 187 and 193 upon applying the tuning voltage of 5, 10, 15, 20, 25 and 30 V, respectively. The simulated results were in agreement with the measured results. Both of results showed that the resonant frequency of the resonator increased as the tuning voltage increased.

Figure 14 shows the measured and simulated results for the resonant frequency of the tunable resonator at the different tuning voltages, where the measured data are from Figure 13. The measured results showed that the resonant frequency changed from 183 kHz to 197 kHz when the tuning voltage increased from 0 V to 30 V, an increase of 14 kHz with a tuning voltage of 30 V. Therefore, the maximum frequency-tuning range of the resonator was about 7.6%. As shown in Figure 14, when the tuning voltage increased from 0 V to 30 V, the simulated results presented that the resonant frequency changed from 173 kHz to 193 kHz, and the measured results showed that the resonant frequency varied from 183 kHz to 197 kHz. The error percentage of both results was below 5.8%, which was small and reasonable, and was the reason that the surface of the structural layers had a little residual oxide resulting in the increment of the stiffness and the resonant frequency.

The tunable resonator reported by Yao *et al.* [3], had a driving voltage of 35 V. The driving voltage of the tunable resonator, manufactured by Dai *et al.* [7], was about 20 V. Dai *et al.* [8] proposed a plane resonator that the driving voltage was about 60 V. Comparing with the above literature, the driving voltage of this work was lower than that of Yao *et al.* [3] and Dai *et al.* [7,8].

## 5. Conclusions

The tunable in-plane resonator has been fabricated using the commercial 0.35 μm CMOS process and post-CMOS process. The area of the resonator was about 460×260 μm^2^. The post-process required only one maskless wet etching to remove the sacrificial layer and released the suspended structures of the resonator. The advantages of the post-process included low cost and easy execution. The structures of the tunable resonator consisted of driving, sensing, and tuning parts. The sensing part generated a change in capacitance when applying a voltage to the driving part, and the sensing circuitry was employed to convert the capacitance variation of the sensing part into the output voltage. The resonator needed only a low driving voltage of 10 V. Experimental results revealed that the tunable resonator had a resonant frequency of 183 kHz (which could be tuned by the tuning part), and a Q-factor of 400. The resonant frequency changed from 183 kHz to 197 kHz when the tuning voltage changed from 0 V to 30 V. The maximum frequency-tuning ratio of the tunable resonator was approximately 7.6%.
